# DAILY AFFECTIVE REACTIVITY ACROSS THE COVID-19 PANDEMIC: THE MODERATING ROLES OF POSITIVE AFFECT AND AGE

**DOI:** 10.1093/geroni/igae098.2703

**Published:** 2024-12-31

**Authors:** Niccole Nelson, Cindy Bergeman

**Affiliations:** Colorado State University, Fort Collins, Colorado, United States; University of Notre Dame, Notre Dame, Indiana, United States

## Abstract

On March 11, 2020, the World Health Organization declared COVID-19 a pandemic. Given COVID-19 had the capacity to make any individual seriously ill, several emergency directives, including cancelation of social gatherings and closures of non-essential businesses, were put in place to slow its spread. Individuals over the age of 60 were considered high-risk for serious COVID-19-related illness, which was associated with greater mitigation efforts and lifestyle changes among this group. Although prior work indicates that the emergency phase of the pandemic influenced stress-and-coping processes, less is known about age differences in these processes, especially how they have unfolded across the past four years of the pandemic. Thus, leveraging data from the Notre Dame Study of Health & Well-being—COVID Study, we examined daily stress and affect across five 28-day, daily diary bursts that were collected approximately every six months between April 2020 and February 2022. Using three-level, multilevel modeling, we examined daily negative affective reactivity, the ‘undoing’ effect of daily positive affect on daily negative affective reactivity, as well as age differences in these daily processes. We found that individuals experienced greater negative affect on days of particularly high stress, and that this process was buffered on days of particularly high positive affect. We also found that older individuals exhibited both lower affective reactivity to stress, as well as a greater ‘undoing’ effect, than younger adults. These results indicate that the mobilization of positive affect may be a promising avenue for intervention in daily stress processes, especially among older adults.

